# Blockade of fibroblast activation protein in combination with radiation treatment in murine models of pancreatic adenocarcinoma

**DOI:** 10.1371/journal.pone.0211117

**Published:** 2019-02-06

**Authors:** Andrew J. Gunderson, Tomoko Yamazaki, Kayla McCarty, Michaela Phillips, Alejandro Alice, Shelly Bambina, Lauren Zebertavage, David Friedman, Benjamin Cottam, Pippa Newell, Michael J. Gough, Marka R. Crittenden, Pieter Van der Veken, Kristina H. Young

**Affiliations:** 1 Earle A. Chiles Research Institute, Robert W. Franz Cancer Center, Providence Cancer Institute, Portland, OR, United States of America; 2 Gastrointestinal & Minimally Invasive Surgery, The Oregon Clinic, Portland, OR, United States of America; 3 Radiation Oncology, The Oregon Clinic, Portland, OR, United States of America; 4 Medicinal Chemistry (UAMC), Department of Pharmaceutical Sciences, University of Antwerp (UA), Antwerp, Belgium; Indiana University School of Medicine, UNITED STATES

## Abstract

Pancreatic ductal adenocarcinoma (PDAC) is characterized by a fibrotic stroma with a poor lymphocyte infiltrate, in part driven by cancer-associated fibroblasts (CAFs). CAFs, which express fibroblast activation protein (FAP), contribute to immune escape via exclusion of anti-tumor CD8^+^ T cells from cancer cells, upregulation of immune checkpoint ligand expression, immunosuppressive cytokine production, and polarization of tumor infiltrating inflammatory cells. FAP is a post-proline peptidase selectively expressed during tissue remodeling and repair, such as with wound healing, and in the tumor microenvironment by cancer-associated fibroblasts. We targeted FAP function using a novel small molecule inhibitor, UAMC-1110, and mice with germline knockout of FAP and concomitant knock-in of E. coli beta-galactosidase. We depleted CAFs by adoptive transfer of anti-βgal T cells into the FAP knockout animals. Established syngeneic pancreatic tumors in immune competent mice were targeted with these 3 strategies, followed by focal radiotherapy to the tumor. FAP loss was associated with improved antigen-specific tumor T cell infiltrate and enhanced collagen deposition. However, FAP targeting alone or with tumor-directed radiation did not improve survival even when combined with anti-PD1 therapy. Targeting of CAFs alone or in combination with radiation did not improve survival. We conclude that targeting FAP and CAFs in combination with radiation is capable of enhancing anti-tumor T cell infiltrate and function, but does not result in sufficient tumor clearance to extend survival.

## Introduction

Pancreatic ductal adenocarcinoma (PDAC) is an aggressive malignancy with a poor prognosis characterized by a fibrotic stroma and poor immune infiltrate. PDAC is relatively radioresistant with poor drug penetrance and elevated levels of hypoxia limiting the efficacy of chemoradiotherapy[1]. Radiation therapy is a targeted cytotoxic modality; however, its efficacy can be limited in part by contributions from the tumor stroma. An additional benefit of radiation is its ability to expose tumor antigen and create a focal inflammatory response[2–4]. The efficacy of high-dose radiation is in part dependent on CD8^+^ T cells[1,5,6]. Therefore, radioresistance can be driven by components in the tumor stroma resulting in neovascularization creating hypoxic regions and alterations in the immune environment impairing CD8^+^ T cell infiltration and function.

Fibrosis driven by primarily by cancer-associated fibroblasts (CAFs) may be the link between hypoxia and impaired CD8^+^ T cell infiltration and function. Given the dependence of high-dose radiation on CD8^+^ T cells, combination radiation with immunotherapy has been attempted to enhance PDAC tumor clearance, but has had little success, in part attributed to impaired ability of immune cells to penetrate the fibrotic stoma and interact with tumor cells[1,7,8]. CAFs are key mediators of the fibrotic stroma and mouse models targeting CAFs resulted in improved drug penetrance and CD8^+^ T cell infiltration[9]. However, tumor infiltrating T cells have impaired effector function due to upregulation of immune checkpoint ligand expression on CAFs and other stromal cells[10,11]. CAFs polarize the tumor immune cells to an immunosuppressive phenotype characterized by arginase expressing M2 macrophages and regulatory T cells, as a result of CAF production of IL-6, IL-10, and TGFβ[12–14]. PDACs express high levels of CAF-related fibroblast activation protein (FAP) compared to normal pancreas[15]. FAP is a post-proline peptidase with both exopeptidase and endopeptidase activity, with many unknown substrates, though collagen is amongst its putative targets. Recent data suggests FAP contributes to a suppressive tumor microenvironment via cleavage of collagen to reveal Scavenger Receptor A-2 binding sites for tumor-associated macrophages [16]. FAP is undetectable in most normal tissues, but expressed at sites of tissue remodeling and repair including wound healing, cirrhotic liver, inflamed synovial tissue, and CAFs[17]. FAP provides a more specific marker of CAFs than αSMA and vimentin, and has been used for CAF depletion strategies. Depletion of CAFs by targeting FAP, resulted in improved antitumor immunity characterized by higher levels of IFN-γ and TNFα[18] and enhanced CD8^+^ T cell function and persistence associated with decreased desmoplastic stroma[19–21]. Additionally, in a melanoma tumor model, administration of a vaccine targeting tumor antigen and FAP resulted in tumor clearance as a result of antigen spreading[22]. These studies demonstrate that CAFs contribute to impaired CD8^+^ T cell function. In contrast to the enhanced anti-tumor effect observed in these studies, depletion of CAFs by targeting the less specific CAF marker, αSMA, resulted in enhanced tumor growth and immunosuppression via enhanced regulatory T cells [23]. Similarly, they found that patients whose PDACs expressed less αSMA, indicative of fewer CAFs, had reduced survival [23], suggesting that CAF depletion could have a deleterious effect on patient survival. Due to the contradictory results of CAF depletion, we suggest to target the suppressive functions of CAFs rather than deplete the CAFs. We believe FAP contributes to the suppressive function of CAFs. Recent data suggests that FAP+ CAFs contribute to T cell exclusion at the periphery of tumors and suppression T cell proliferation in a nitric oxide dependent manner[24]. Germline knockout of FAP resulted in increased disorganized collagen deposition in colorectal and pancreatic tumors with diminished tumor growth via indirect effects on tumor cell proliferation and decreased neovascularization[25]. Given the dependence of radiation on CD8^+^ T cells and oxygenation, we hypothesize that FAP function mediates radiation resistance by organization of ECM components restricting T cell infiltration and suppressive polarization of the immune microenvironment via recruitment of M2 macrophages and limiting infiltrating T cells; and via neovascularization mediating hypoxia.

We hypothesize that PDAC resistance to radiation is driven by immunosuppression and hypoxia as a result of the fibrotic stroma orchestrated by CAFs. We believe that FAP function is required for deposition and organization of extracellular matrix components, including collagen, and provides a target for improving tumor oxygenation and the quantity and function of the immune infiltrate. To date, the combination of radiation and FAP inhibition has not been evaluated. Herein, we evaluate a novel selective small molecule inhibitor of FAP[26,27], germline knockout of FAP, and adoptive transfer of T cells that recognize CAFs to enhance the immune effects of high dose radiation in an immunocompetent murine model of PDAC.

## Materials/Methods

### Animals and cell lines

The Panc02 murine pancreatic adenocarcinoma cell line[28], was kindly provided by Dr. Woo (Mount Sinai School of Medicine, NY). Panc02-SIY cell line was provided by R. Weischelbaum. Cells were grown in DMEM media supplemented with HEPES, non-essential amino acids, sodium pyruvate, glutamine, 10% FBS, penicillin and streptomycin. All cell lines tested negative for mycoplasma. *C57BL/6* and FAP^LacZ^ knockout mice were obtained from Jackson Laboratories (Bar Harbor, ME). MMTV-PyMT mice were backcrossed onto C57BL/6 for >10 generations and previously described[29]. Gender matching of control and treatment groups was attempted for all experiments. Tumor bearing mice were monitored a minimum of three days per week and euthanized when tumors exceeded 12 mm in any dimension, or when body condition score declined one level. Euthanasia was performed with CO_2_ inhalation followed by a second method, either organ harvest or cervical dislocation. Radiation was performed with inhaled isoflurane anesthesia and intraperitoneal meloxicam was given for analgesia. There were no unexpected animal deaths. All animal protocols were approved by the Earle A. Chiles Research Institute IACUC (Animal Welfare Assurance No. A3913-01).

### Antibodies and reagents

UAMC-1110 was generously provided by Dr. Pieter Van der Veken (University of Antwerp). Unless otherwise specified, UAMC-1110 was administered in high molecular weight PEG at a concentration of 20mg/kg by oral gavage twice per day. Fluorescently-conjugated antibodies CD3-e450, CD8-PerCP, CD4-FITC, CD4-e450, CD4-PerCP, CD25-APC, IFNγ-APC, IL-2-PE, TNFa-PE-Cy7, CD11b-PE-Cy7, Ly6G-FITC, Ly6C-PerCP-Cy5.5, Gr1-PE-Cy7, and MHCII-EF450, PDGFRβ-APC, CD31-PE, CD45-BV510, and EpCam-BV605 were purchased from Thermo Fisher Scientific (Lafayette, CO) or BD Biosciences (San Jose, CA). CD8-PE-TxRD was purchased from Invitrogen (Carlsbad, CA). SIY, SIINFEKL, and β-galactosidase peptides were obtained from Integrated DNA Technologies (Coralville, Iowa). β-galactosidase (βgal) for the ICPMYARV peptide and SIINFEKL peptide tetramers were obtained from the NIH Tetramer core facility (Atlanta, GA). Antibodies to CD31 (Thermo Fisher Scientific), αSMA (Sigma-Aldrich, St Louis, MO), PDGFRα (Thermo Fisher Scientific), Vimentin (Cell Signaling Technology, MA), Ki67 (Abcam), Cleaved Caspase 3 (Cell Signaling Technology) were used for immunofluorescent staining and hypoxyprobe Omni Kit (Burlington, MA) was used for hypoxia staining. Anti-PD1 antibody clone RPM1-14 (BioXcell, West Lebanon, NH) was used where indicated for treatment studies.

### Listeria vaccination

A gene containing the sequence of the actA gene fused to the sequence coding for the desired epitopes was synthesized at Integrated DNA Technologies (Coralville, Iowa) in an IDT Blunt Vector with Kanamycin resistance. The vector was used as a source for the DNA sequence to be cloned into the pPL2e vector and conjugated into a Listeria monocytogenes strain. Immunodominant βgal Kb epitopes selected for expression were amino acids 96–103: DAPIYTNV and 497–504: ICPMYARV. Internal control for confirming vaccination was the ovalbumin SIINFEKL peptide. For *in vivo* use, LM vaccines were washed and resuspended in PBS for injection. A dose of 5x10^6^ -1x10^7^ CFU was delivered intravenously and confirmed by plating of residual inoculum.

### *In vitro* co-cultures

CD8^+^ T cells were purified using a cocktail of biotin conjugated, lineage marker antibodies not expressed by CD8^+^ T cells and negatively selected using streptavidin conjugated magnetic beads on an AutoMACS systems (Miltenyi Biotech). Purified CD8^+^ T cells (3x10^5^) were cultured alone, with plate bound anti-CD3/anti-CD28 (5 μg/ml), or 6x10^4^ of either MCA-OVA cells, Panc02 tumor cells, C57BL/6J mouse fibroblasts, or fibroblasts pretreated with TGFβ (1 ng/ml) for 8 hours. Following 18 hours of culture, cells were supplemented with Brefeldin A to stop golgi export of cytokines for 5 hours. Cells were harvested for intracellular FACS analysis and percent viable tumor cells or fibroblasts compared to non co-culture groups alone. A portion of the purified CD8^+^ T cells was reserved for FACS staining and confirmation of purity which was routinely greater than 95% viable CD8^+^ T cells.

### *In Vivo* radiotherapy models

2x10^5^ Panc02 and 5x10^6^ Panc02-SIY cells were injected in 100 μL of PBS or serum free DMEM subcutaneously in the right flank of immunocompetent *C57BL/6* mice. PyMT-MMTV tumors were harvested from d100 animals on a *C57BL/6* background, digested into a single cell suspension, pooled, and frozen. PyMT-MMTV cells were injected into the mammary fat pad of immunocompetent *C57BL/6* or FAP KO mice. For radiation treatment, mice were anesthetized by isoflurane inhalation on the stage of a Small Animal Radiation Research Platform (SARRP, XStrahl, GA), and CT imaged. Dosimetry was performed using SLICER software with SARRP-specific add-ons (XStrahl) and treatment calculated to an isocenter in the tumor target. A single beam at 45 degrees using a 5x5 mm collimator was utilized to deliver 10 Gy x 3 (Panc02, Panc02-SIY) or 10 Gy x 1 (PyMT-MMTV) beginning on day 14.

### Flow cytometry

For analysis of tumor infiltrating immune cells, the tumor was digested in 10 mL of PBS with 1 mg/mL collagenase (Thermo Fisher Scientific), 100 μg/mL hyaluronidase (Sigma-Aldrich), and 50 kU/ml DNase I (Sigma-Aldrich) for up to 1hr at room temperature. Single cell suspensions were filtered through 100μm nylon mesh and stained with antibodies specific for surface antigens, then washed and fixed using a T regulatory cell staining kit (Thermo Fisher Scientific) and intracellularly stained for FoxP3 as previously described[30]. Spleens and lymph nodes were crushed, filtered and stained as above. The proportion of each infiltrating cell type was analyzed on a BD LSRII.

### Immunohistochemistry

Tumors were fixed in Z7 zinc based fixative[31] overnight. Tissue was then processed for paraffin tissue sections. Tissue was dehydrated through graded alcohol to xylene, incubated in molten paraffin using a Tissue-Tek automated tissue processor (Sakura, Torrance, CA), and then embedded in paraffin. 5 μm sections were cut and mounted for analysis. Trichrome stain was performed according to manufacturer’s protocol (Cardinal Health, Dublin, OH). Primary antibody binding was visualized with Alexa Fluor 488 (Thermo Fisher Scientific), Opal 520, or Opal 620 secondary antibodies (Perkin Elmer, Boston, MA) following either ImmPRESS HRP anti-rabbit or anti-rat polymer incubation (Vector laboratories). Sections were stained with DAPI (Perkin Elmer) for nuclear staining and mounted with FluoromountG (Thermo Fisher Scientific). Images were acquired using: Vectra imaging software (Perkin Elmer); a Leica SCN400 whole slide scanner or a SCN400F fluorescence whole slide scanner. All images displayed in the manuscript are representative of the entire tumor and their respective experimental cohort. NIH image J software was used to separate into their single marker components, to set threshold to minimize back ground noise, and to quantify the positive pixel area in each image. Minimum of 4 tumors per cohort were utilized for analysis.

### Expression analysis

Publically available RNASeq data from TCGA using cBioportal was queried for FAP expression in all tumor types. Publically available microarray data from GEO Accession #GSE15471 was queried for differential expression of FAP in pancreatic ductal adenocarcinoma compared to patient matched normal pancreatic tissue expression.

### Statistics

Data were analyzed and graphed using Prism (GraphPad Software, La Jolla, CA). Individual data sets were compared using Student’s T-test and analysis across multiple groups was performed using ANOVA with individual groups assessed using Tukey’s comparison. Tumor growth curves were compared using linear regression curve fitting and performing pairwise comparison of slope. Kaplan and Meier survival curves were compared using a log-rank test.

## Results

### FAP inhibition alters tumor microenvironment

**Fig 1:**
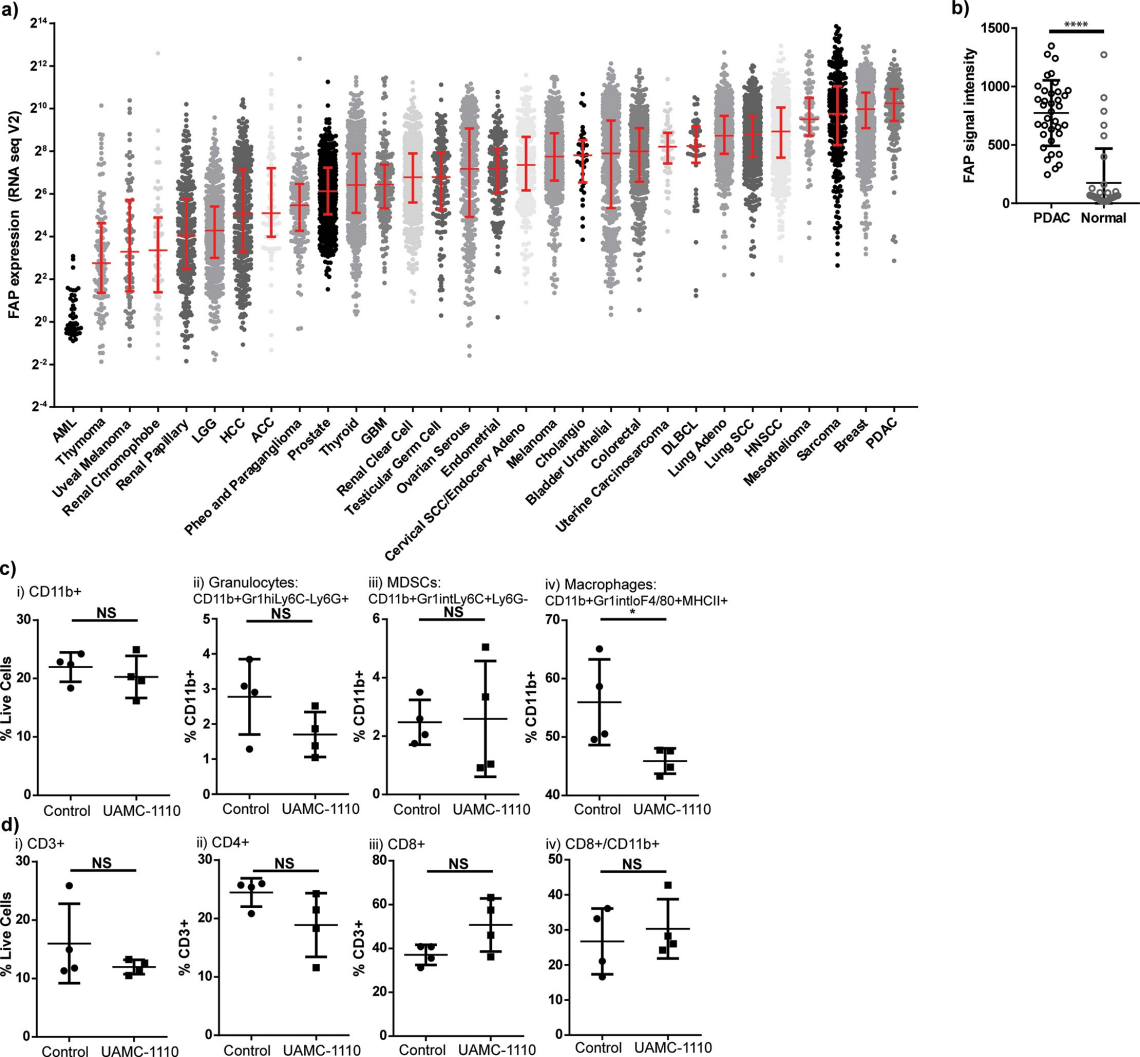
Fibroblast activation protein expression elevated in PDAC contributes to tumor infiltrating macrophages. a) FAP expression by RNAseq in human tumor types queried through TCGA database. b) FAP expression in normal pancreas and PDAC samples from GEO Accession database. Panc02 pancreatic adenocarcinoma tumor infiltrating c) CD11b^+^ and d) T cells by flow cytometry in control vs FAP inhibitor (UAMC-1110) treated animals. Treatment delivered days 7–13, tumors harvested day 14. n = 4–6 female mice/group, representative experiment. NS = not significant, *p<0.05.

To determine the most clinically appropriate tumor type in which to study the CAF marker, fibroblast activation protein (FAP), we queried The Cancer Genome Atlas (TCGA) database for FAP expression in all available tumor types. We found that the median expression of FAP was highest in pancreatic ductal adenocarcinoma (Fig 1A). We subsequently queried the relative expression compared to normal pancreas tissue using a publically available GEO Accession dataset obtained from whole tissue microarray expression from 36 paired PDACs and normal pancreas tissue. We found the FAP mean signal intensity in PDAC was 772 versus 174 in normal pancreas (p<0.0001, Fig 1B). Therefore, we proceeded to evaluate the role of FAP in murine models of PDAC treated with radiation to model this unique human tumor biology.

**Fig 2:**
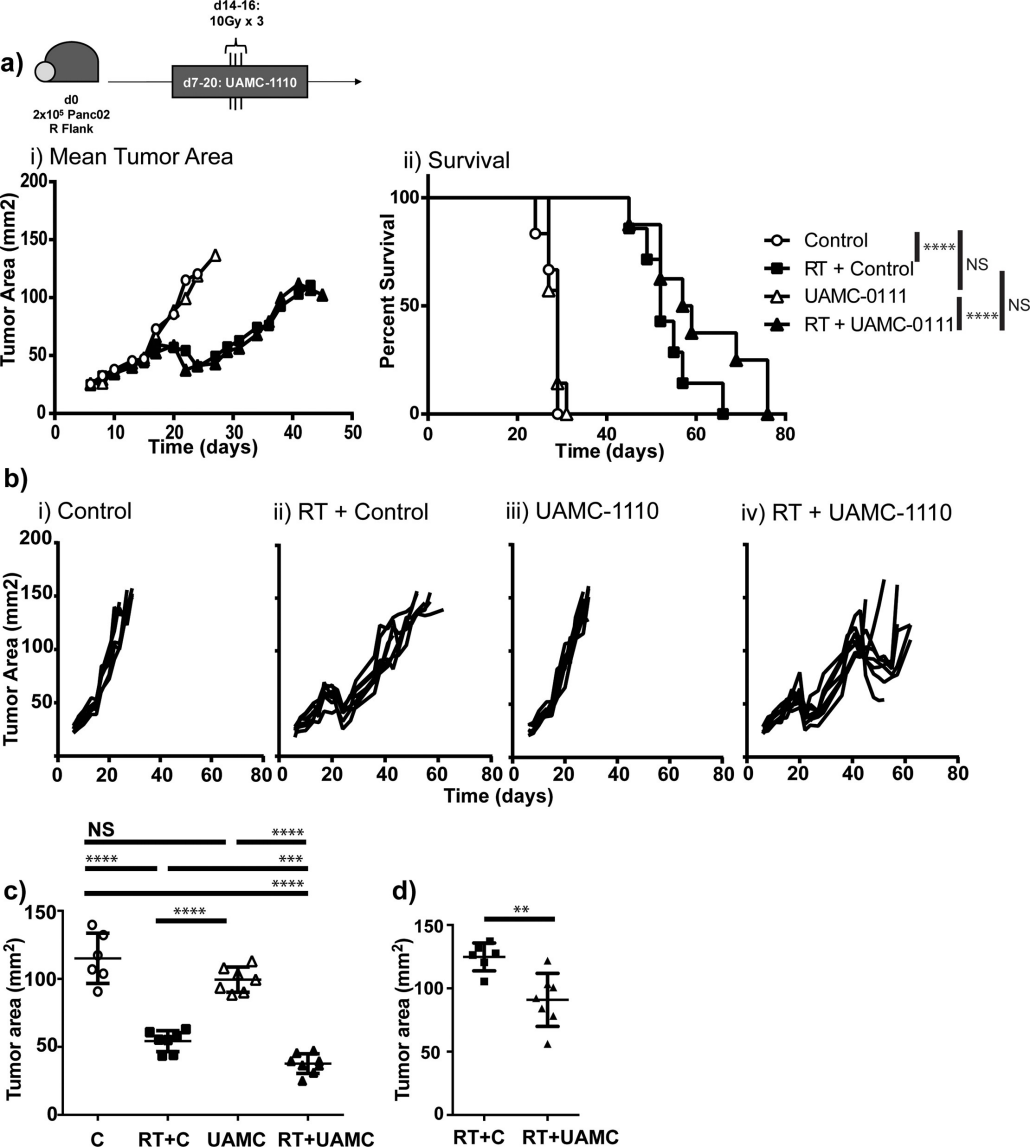
Tumor growth delay with FAP inhibitor. Panc02 tumor bearing mice treated with vehicle control (Control) or FAP inhibitor (UAMC-1110) days 7–21, randomized to receive 10 Gy x 3 tumor directed radiation (RT) days 14–16. a) i) Mean tumor area and ii) survival curves. b) Individual tumor growth curves. c) Day 23 tumor measurements. d) Day 48 tumor measurements. n = 6–8 female mice/group, representative experiment. NS = not significant, **p<0.01, ***p<0.001, ****p<0.0001.

We injected immunocompetent *C57BL/6* mice with 2x10^5^ Panc02 syngeneic pancreatic ductal adenocarcinoma tumor cells on day 0. Mice were treated with vehicle control or a novel small molecule inhibitor of FAP (UAMC-1110)[26,27] beginning on day 7 and administered to day 13 and tumors were harvested on day 14. Mice that received UAMC-1110 had fewer tumor infiltrating macrophages compared to control treated animals (Fig 1Civ), but no differences were observed in other infiltrating immune cells (Fig 1C and 1D). Recently published data suggests that FAP-mediated cleavage of collagen can be a substrate for binding of tumor associated macrophages scavenger receptor-A[16]. Our results are consistent with this finding, in that FAP inhibition was associated with fewer tumor-infiltrating macrophages. We evaluated the effect of FAP inhibition on CAF numbers and found that blockade of FAP function did not alter the number of CAFs in the tumor by IHC or FACS (S1 Fig). We have previously demonstrated that macrophages can limit radiation responses in PDAC[32], therefore we hypothesized that FAP inhibition would improve radiation responses. To test this hypothesis, we treated Panc02 tumor bearing mice with UAMC-1110 on days 7–21 and randomized them to receive sham vs 10 Gy x 3 tumor targeting radiation on days 14, 15, and 16, using the Small Animal Radiation Research Platform (SARRP) to minimize dose to surrounding structures. We found that addition of UAMC-1110 to radiation resulted in a mild tumor growth delay at day 23 (Fig 2A–2C) and a more marked growth delay at day 48 (Fig 2Biv and 2D). However, despite growth delays, tumors continued to progress resulting in euthanasia due to tumor size, without a significant improvement in overall survival (Median Survival 52d vs 58d, p = 0.09, Fig 2wAii). These data suggest that short-term FAP inhibition combined with tumor-directed radiation was sufficient to delay tumor growth, but extended survival might require more prolonged or complete inhibition of FAP function.

**Fig 3:**
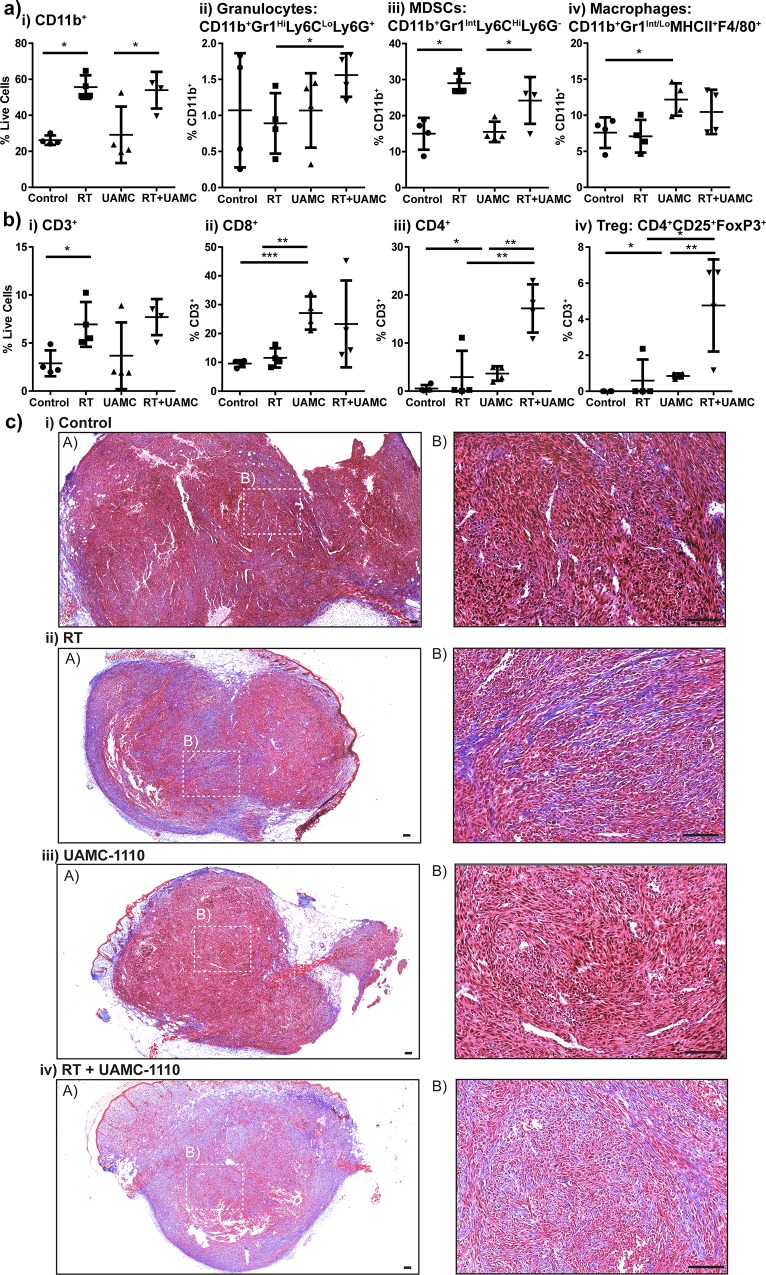
FAP inhibition and radiation alter tumor microenvironment. Panc02 tumor bearing mice treated with vehicle control (Control) or FAP inhibitor (UAMC-1110) days 7–21, randomized to receive 10 Gy x 3 tumor directed RT days 14–16. Tumor infiltrating a) CD11b^+^ cells and b) T cells on day 23. c) Trichrome stain at 4x (A) and 20x (B), 100 μm scale bar in c. Selected images are representative of group. n = 4–6 female mice/group. NS = not significant, *p<0.05, **p<0.01, ***p<0.001.

To determine how FAP inhibition combined with radiation resulted in tumor growth delay, we interrogated the tumor microenvironment at time points where we observed differences in tumor growth. The animals were treated as described above, and tumors were harvested on day 23, two days following completion of UAMC-1110 therapy and one week following last dose of radiation, and day 43. Tumors from mice treated with both UAMC-1110 and radiation demonstrated differences in tumor immune infiltrate and collagen deposition (Fig 3). Radiation increased tumor infiltrating CD11b^+^ cells, predominately within the Gr1^INT^ Ly6C^HI^ Ly6G^-^ population, also known as immature monocytes or myeloid-derived suppressor cells (MDSCs) (Fig 3Aiii). While we observed a decrease in tumor infiltrating macrophages during administration of UAMC-1110 (Fig 1C iv), we found withdrawal of UAMC-1110 led to a rebound in macrophage infiltrate (Fig 3Aiv). We also observed an increase in tumor infiltrating CD3^+^ T cells in mice receiving radiation (Fig 3Bi), consistent with literature[5,33]. UAMC-1110 significantly increased tumor infiltrating CD8^+^ T cells, including effector subpopulations (Fig 3Bii and data not shown), while combination UAMC-1110 and radiation increased CD4^+^ T cells, including regulatory T cells (Fig 3Biii-iv). Despite increased CD8^+^ T cell infiltrate, there were still only approximately 3 CD8^+^ T cells per 100 CD11b^+^ cells in day 23 tumors (data not shown). To evaluate whether desmoplastic stroma might be physically excluding tumor immune cell infiltrate, we analyzed tumor collagen deposition by trichrome staining (Fig 3C). Radiation increased tumor collagen, as did UAMC-1110 to a lesser degree, and the combination of UAMC-1110 and radiation increased collagen further above that seen with radiation alone (Fig 3Ciii-iv). This is consistent with previous data demonstrating FAP loss is associated with increased, but disorganized collagen deposition[25]. Evaluation of the tumor immune infiltrate at day 43, during the second tumor growth delay, did not result in any significant difference in tumor immune infiltrate or collagen deposition (data not shown). We further evaluated tumor cell proliferation and apoptosis at days 23 and 43 and found no difference with the addition of UAMC-1110, however there was a significant increase in both proliferation and apoptosis between UAMC-1110 and radiation treated tumors at day 43 compared to day 23 (S1F, S1G, S1I and S1J Fig). These data suggest that short duration of FAP inhibition was insufficient to prolong survival, possibly due to withdrawal of drug resulting in rebound macrophage infiltration into the tumor and functional recovery of collagen cleavage by FAP leading to matrix re-organization and tumor contraction in size, but a rebound in tumor cell proliferation and no improvement in immune mediated tumor clearance. Therefore, we hypothesized that prolonged FAP inhibition combined with radiation would be more effective. We evaluated the effect of radiation in mice with germline knockout of FAP and knock-in of E. coli βgal driven by the FAP promoter[34].

### FAP knockout mice demonstrate alterations in immune responses

**Fig 4:**
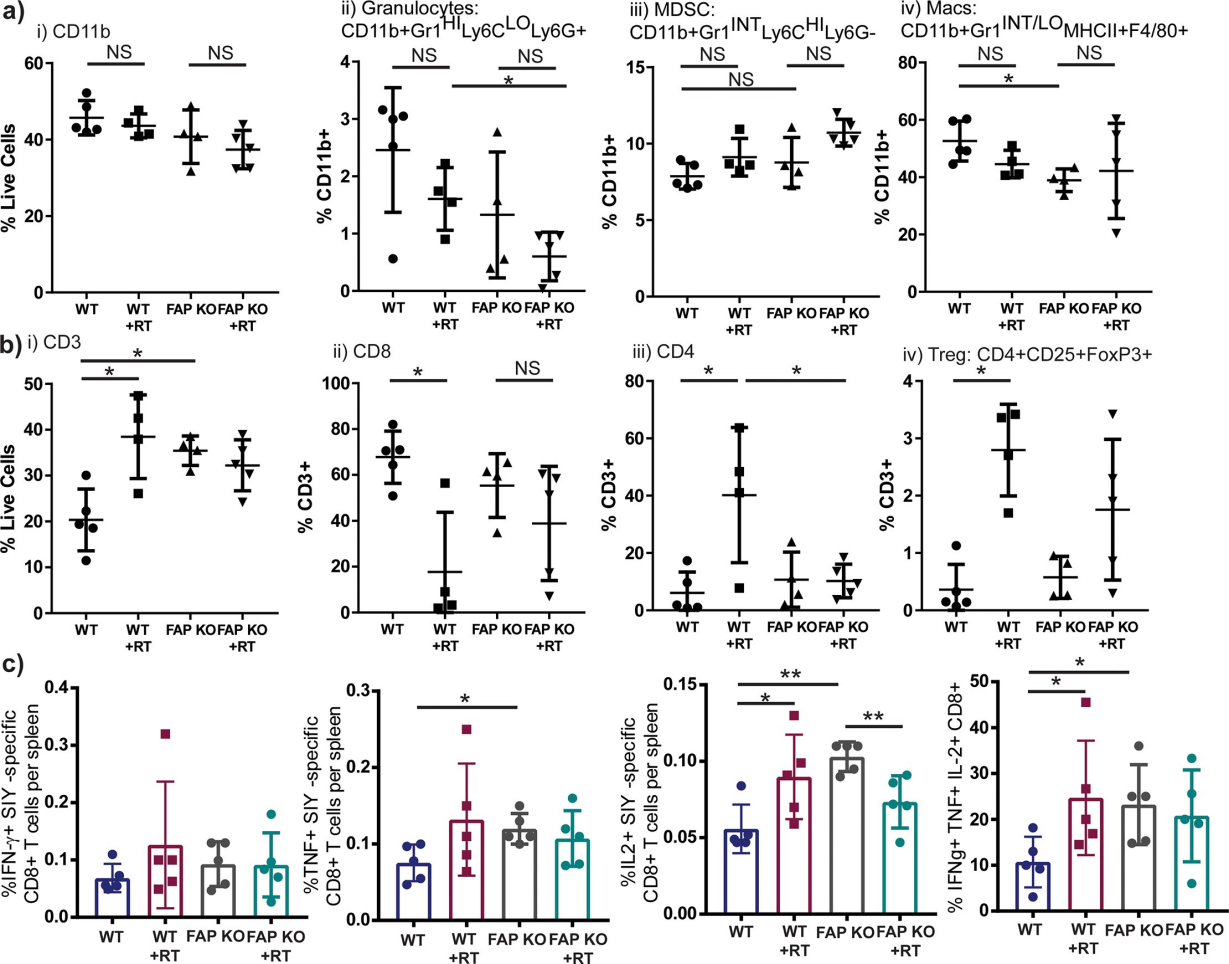
FAP KO or radiation results in enhanced T cell tumor infiltrate. Panc02-SIY tumor bearing mice in WT (WT) or FAP knockout (FAP KO) animals, randomized to receive 10 Gy x 3 tumor directed RT days 14–16. Tumor infiltrating a) CD11b^+^ and b) T cells on day 23. c) Splenocytes stimulated *ex-vivo* with SIY peptide from tumor bearing mice and assessed for intracellular IFN-γ, TNFα, and IL-2 by flow cytometry. n = 4–6 mixed gender mice/group. NS = not significant, *p<0.05, **p<0.01.

In order to test a model with complete and permanent loss of FAP, we injected WT *C57BL/6* mice (WT) and FAP knockout (FAP KO) animals in a *C57BL/6* background with Panc02 PDAC cells which express the model tumor antigen SIYRYYGL (Panc02-SIY), to allow tracking of antigen-specific immunity, on day 0. Mice were randomized to sham versus 10 Gy x 3 tumor directed radiation on days 14–16. Mice were then euthanized on day 23 and tumors and spleens were harvested. FAP KO animals demonstrated fewer tumor macrophages (Fig 4ivA). In addition, tumors from FAP KO animals demonstrated more CD3^+^ T cells at baseline, but did not exhibit a further increase following radiation (Fig 4Bi). While WT animals had fewer CD3+ T cells at rest, they demonstrated an increase following RT to levels equivalent to FAP KO animals (Fig 4Bi). Radiation specifically increased the proportion of tumor infiltrating CD4^+^ T cells, including regulatory T cells in WT mice (Fig 4Biii-iv), with a reciprocal decrease in the proportion of tumor infiltrating CD8^+^ T cells, including effector subtypes (Fig 4Bii and data not shown). To evaluate antigen-specific T cell responses, we evaluated intracellular cytokine responses to *ex-vivo* stimulation with SIY peptide from splenic lymphocytes derived from tumor bearing mice. We found that SIY-stimulated splenocytes from FAP KO animals demonstrated enhanced TNFα and IL-2 production compared to WT animals (Fig 4C). Radiation enhanced IL-2 and polyfunctional splenocyte responses in WT animals, but not in FAP KO animals (Fig 4C). These data suggest that loss of FAP or radiation treatment alone are capable of enhancing anti-tumor T cell function, but not in an additive or synergistic fashion. Next, we evaluated whether tumor cytokines were different between WT and FAP KO tumors, and whether radiation influenced tumor cytokines (S2 Fig). Tumors from FAP KO animals had less FGF, while no differences were observed in TNFα, IFN-γ, or VEGF (S2 Fig). IL-2 and MIP-1α were increased in FAP KO animals following radiation compared to WT mice receiving radiation (S2 Fig). These data demonstrate that tumors in FAP KO mice exhibit a distinct immune profile in their tumor resulting in improved antigen-specific responses systemically.

**Fig 5:**
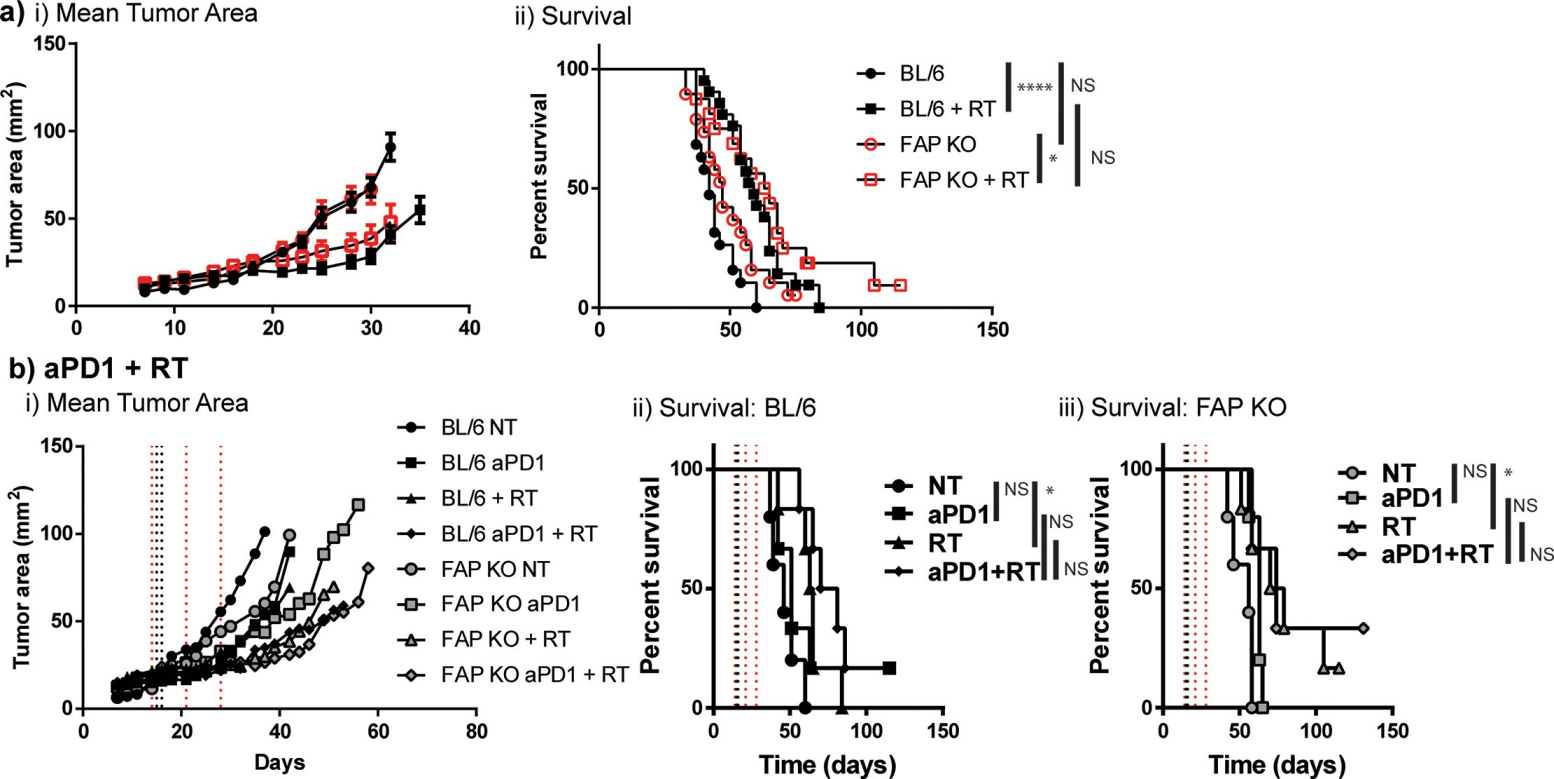
Radiation does not improve survival when targeting FAP. a) Panc02-SIY tumor bearing mice in WT or FAP KO animals, randomized to receive 10Gy x 3 tumor directed RT days 14–16. n = 12–18 mixed gender mice/group. b) Same treatment as in (a) +/- anti-PD1 days 14, 21, and 28. n = 6–8 mixed gender mice/group. NS = not significant, *p<0.05, ****p<0.0001.

To evaluate whether the alterations in tumor immune infiltrate, namely decreased macrophages and increased CD3^+^ T cells, with enhanced cytokine production upon antigen stimulation, translate into improved tumor growth and survival, we treated mice as described above (Fig 4). We observed no difference in tumor growth or survival in FAP KO compared to WT mice (Median survival 47d vs 42d, Fig 5Ai-ii). We also observed that radiation improved survival in WT mice (median survival 59d, p<0.0001, Fig 5Aii), and FAP KO mice (median survival 64d, p = 0.03, Fig 5Aii), but as with FAP inhibitor (Fig 2A ii), no additional benefit to radiation in FAP KO mice over WT mice. To evaluate whether vascular alterations or the increased disorganized collagen observed in tumors from FAP KO animals contributed to increased hypoxia limiting radiation response, we examined tumor vascularity using CD31 and hypoxia using hypoxyprobe injected into tumor bearing WT and FAP KO animals 14 days after Panc02-SIY tumor challenge, prior to radiation. We observed no differences in hypoxic area or tumor vascularity in FAP KO animals compared to WT controls (S1A–S1D Fig). To determine whether this was tumor model specific, we evaluated orthotopic mammary tumors derived from single cell suspensions of the spontaneously derived mouse mammary tumor virus-polyoma middle T antigen (MMTV-PyMT) transgenic mouse. Tumor digests were implanted (5x10^5^ cells/injection) into the mammary fat pads of WT and FAP KO mice given that FAP expression is elevated in human breast cancer (Fig 1A). There was no difference in tumor growth or response to radiation in this mammary carcinoma model (S3 Fig).

An alternate explanation for the limited radiation response in the FAP KO animals despite improved T cell numbers, was non-functional CD8^+^ T cell responses. We and others have reported that tumor growth delay in response to high-dose radiation, is dependent, in part, on CD8^+^ T cells[1]. To evaluate whether immune checkpoint activation through the PD1 pathway contributed to limiting radiation response, we tested combination anti-PD1 antibody with radiation in WT and FAP KO animals. Panc02-SIY tumor bearing animals were treated with anti-PD1 antibody on day 14, 21, and 28, while 10 Gy x 3 radiation was delivered on days 14, 15, and 16 as above. Of note, PDL1 expression on Panc02 parental and Panc02-SIY tumor cell lines in vitro was minimal at baseline, but significantly increased 24h following radiation or stimulation with IFNγ (S4 Fig), and was universally high by 72h (data not shown). We observed improved survival with radiation compared to no treatment (NT) (median survival WT with NT 46d, WT with radiation treatment (RT) 64d, FAP KO with NT 56d, FAP KO with RT 74.5d, Fig 5B), and single agent anti-PD1 failed to provide a survival benefit over NT (median survival WT with anti-PD1 51d, FAP KO with anti-PD1 63 d, Fig 5B). Addition of anti-PD1 to RT did not improve survival in either WT *C57BL/6* or FAP KO animals (median survival WT 75.5d, FAP KO 74d, Fig 5B). Further, no improvement in survival was seen in parallel groups between WT and FAP KO animals (Fig 5B). Combined, these data suggest that radiation response in this model is not limited by immune checkpoint upregulation in FAP KO animals.

### CAF depletion in FAP KO animals has little effect on survival

**Fig 6:**
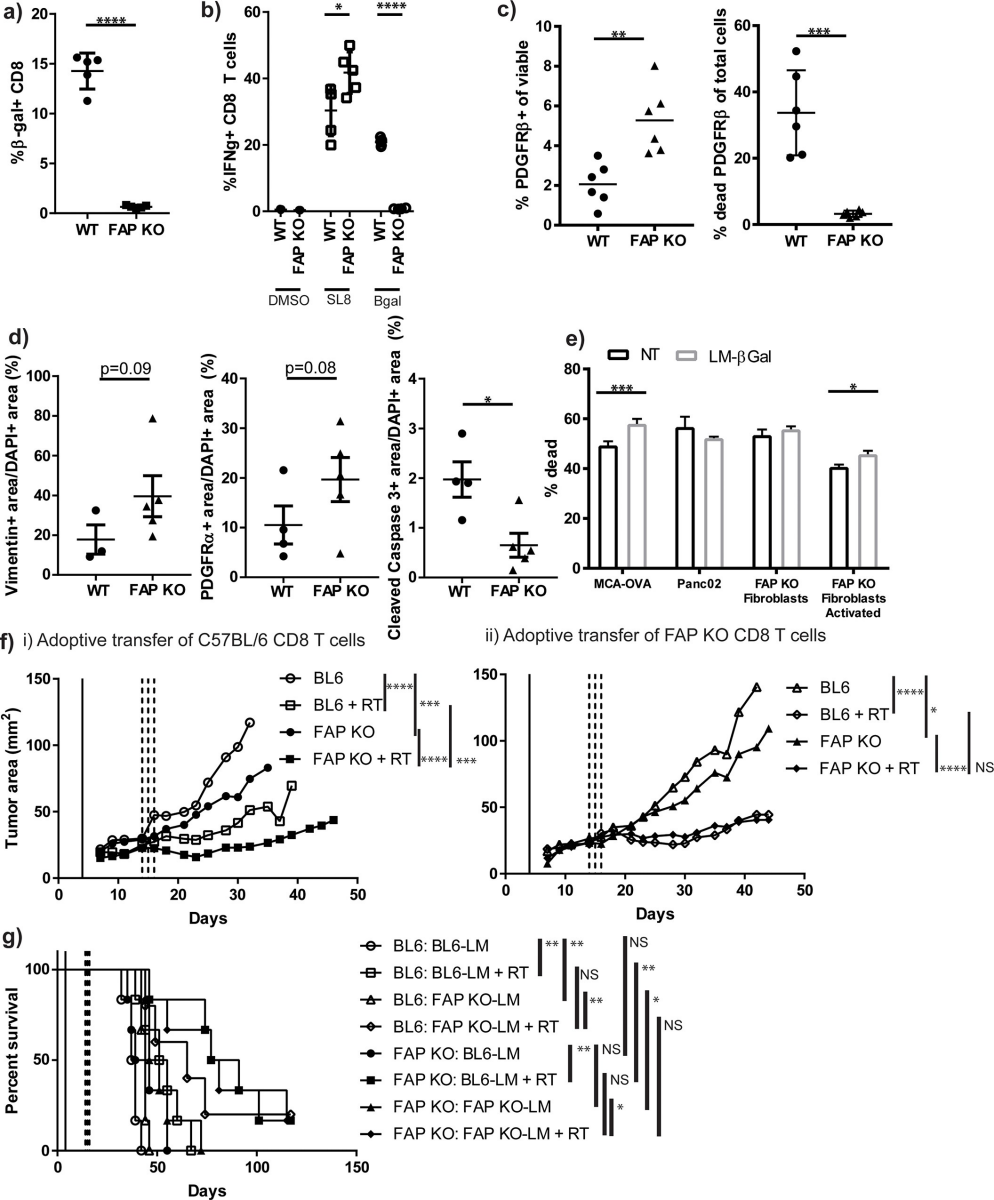
CAF depletion does not enhance radiation efficacy. a) Tetramer positive CD8^+^ T cells in the peripheral blood 7 days after WT or FAP KO mice were vaccinated with an attenuated *Listeria monocytogenes* strain expressing immunodominant βgal peptides 96–103: DAPIYTNV and 497–504: ICPMYARV (LM-βgal) and control OVA peptide SIINFEKL. n = 5 mixed gender mice/group. b) Intracellular IFN-γ^+^ CD8^+^ T cells from splenocytes derived from mice vaccinated with LM-βgal, boost with LM-βgal day 14, and stimulated ex-vivo with peptide: vehicle (DMSO), SL8 (SIINFEKL), or βgal (ICPMYARV). n = 5 mixed gender mice/group. c) Adoptive transfer of 1x10^6^ CD8^+^ T cells from WT or FAP KO LM-βgal vaccinated animals into tumor bearing FAP KO animals on day 4, tumors harvested day 14 and analyzed by flow cytometry for CD45-EpCAM-CD31-PDGFRβ+ cells. n = 6 mixed gender mice/group. d) Quantified immunofluorescence for Vimentin-positive area (left), PDGFRα-positive area (middle), and cleaved caspase-3 positive area (right) per DAPI-positive area. e) Co-culture of tumor cells or primary dermal fibroblasts treated with vehicle or activated with TGFβ, together with splenocytes derived from WT mice 7 days following vaccine with control (NT) or LM-βgal. n = 3 mixed gender mice/group. MCA-OVA = ovalbumin expressing MCA tumor cells. f-g) Adoptive transfer of 1x10^6^ CD8^+^ T cells from WT (i) or FAP KO (ii) LM-βgal vaccinated animals into tumor bearing animals on day 4, 10 Gy x 3 tumor directed RT on days 14–16. Tumor growth in f) and survival in g). n = 6–8 mixed gender mice/group (female transfer into male mice allowed, but no male T cell transfer into female mice). The first genotype listed in the legend in part (g) refers to the tumor-bearing animal. The genotype of transferred T cells is provided after the colon and is hyphenated with “-LM” to designate vaccination with L. monocytogenes. For clarity, group symbols for f and g are the same. RT = radiation. NS = not significant, *p<0.05, **p<0.01, ***p<0.001, ****p<0.0001.

Although FAP may be useful as a marker of CAFs, inhibiting the function of FAP alone was not sufficient to prolong survival in combination with radiation. We evaluated whether knockout of FAP altered the number of CAFs within the tumors. αSMA positive, vimentin positive, and PDGFRα positive tumor area was equivalent between FAP KO tumors and WT tumors (S1C, S1E, S1F, S1H, S1I and S1K Fig) demonstrating equivalent numbers of CAFs. As targeting the function of FAP was not sufficient to improve survival in combination with radiation, we hypothesized that targeting CAF numbers with radiation might improve outcome in PDAC. We targeted fibroblasts using adoptive transfer of antigen-specific T cells against CAFs. We utilized the E. coli βgal expression under the FAP promoter in our FAP KO animals to target CAFs for immune mediated depletion, since in our FAP KO mice, cells that would typically express FAP now express βgal. We generated an attenuated *Listeria monocytogenes* (LM) strain that expressed immunodominant βgal peptides 96–103: DAPIYTNV and 497–504: ICPMYARV (LM-βgal). We vaccinated WT *C57BL/6* and FAP KO mice with LM-βgal, and evaluated tetramer positive CD8^+^ T cells in the peripheral blood 7 days later, and found 14.3% of peripheral blood from WT mice and 0.6% from FAP KO mice exhibited an anti-βgal response (p<0.0001, Fig 6A) demonstrating that the FAP KO mice express βgal, as engineered in the fibroblasts, and are tolerant to the βgal antigens. Following boost with LM-βgal on day 14, we evaluated tetramer positive splenocytes and intracellular cytokine production to peptide stimulation. We observed 20% of CD8^+^ T cells produced IFN-γ in response to peptide stimulation with ICPMYARV in WT *C57BL/6* animals, while only 0.8% IFN-γ^+^ CD8^+^ T cells were observed in the FAP KO animals (p<0.0001, Fig 6B). These data demonstrate the appropriate vaccine response to LM, but tolerance to βgal in the FAP KO mice. To evaluate our hypothesis that immune-mediated clearance of PDAC after radiation would be enhanced following CAF depletion, we adoptively transferred 1x10^6^ CD8^+^ T cells from either WT or FAP KO LM-βgal vaccinated animals, with approximately 10–15% of cells testing for βgal reactivity, into tumor-bearing WT or FAP KO animals on day 4, and tumors were harvested on day 14 and evaluated for CAF depletion. We observed less CAFs in FAP KO animals receiving anti-βgal T cells from WT animals compared to CD8 T cells transferred from vaccinated FAP KO animals (p<0.01, Fig 6C), which corresponded with increased dead CAFs (p<0.001, Fig 6C). This was consistent with changes in CAFs visualized by IHC for Vimentin, PDGFRα, and cleaved caspase 3 (Fig 6D). No difference was observed in proliferation by Ki67 IHC (data not shown). To demonstrate specific T cell activity, we performed in vitro co-culture of splenocytes 7 days after NT or LM-βgal vaccination of WT animals with MCA-OVA as an internal control as our LM-βgal vaccine contains a SIINFEKL peptide, Panc02 tumor cells, FAP KO primary dermal fibroblasts, and FAP KO primary dermal fibroblasts treated with TGFβ to induce activation of β-galactosidase. We observed increased cell death where T cells derived from LM-βgal vaccinated animals were co-cultured with MCA-OVA and activated FAP KO primary dermal fibroblasts (Fig 6E). Animals were vaccinated and CD8 T cells were adoptively transferred into tumor bearing animals as above, followed by radiation on days 14–16. We observed tumor growth delay in the FAP KO animals compared to WT animals receiving anti-βgal T cells (Fig 6Fi, open vs closed circle); however, this did not translate to improved survival (Fig 6G, open vs closed circle, p = 0.12), unless combined with radiation (Fig 6G, open vs closed square, median survival 53 vs 84 days, p<0.01). There was a non-significant trend towards delayed tumor growth in FAP KO mice receiving anti-βgal T cells compared to tolerized T cells from vaccinated FAP KO animals (Fig 6Fi-ii, closed circle vs closed triangle, p = 0.09), which also failed to demonstrate a survival difference (Fig 6G, closed circle vs closed triangle, median survival 42.5 days vs 48.5 days). No difference was observed between FAP KO tumor bearing animals who received radiation when comparing transferred T cells (Fig 6F and 6G, closed square vs closed diamond). However, a significant tumor growth delay was observed in FAP KO tumor bearing animals compared to WT BL/6 tumor bearing animals who received tolerized T cells from vaccinated FAP KO animals (Fig 6Fii, open triangle vs closed triangle, p<0.05), which translated into a survival advantage (Fig 6G, open triangle vs closed triangle, p<0.05). This advantage was not seen with the addition of radiation (Fig 6Fii–6G, open diamond vs closed diamond). Based on these data, we observed tumor growth delay in FAP KO tumor bearing animals compared to WT tumor bearing animals receiving the same T cells suggesting that the FAP KO backgrounds conferred a tumor growth advantage when T cells were transferred into the animals. This may be due to increased infiltration of T cells into the FAP KO tumors leading to a pro-inflammatory cytokine cascade. When comparing FAP KO tumor bearing animals who received anti-βgal T cells to those who received tolerized FAP KO T cells (closed circle vs closed triangle and closed square vs closed diamond), there was no tumor growth delay or survival benefit. These data demonstrate that T cell targeting of CAFs in combination with radiation was not superior to radiation alone suggesting further suppressive factors limit tumor clearance.

## Discussion

Hallmarks of immune suppression in PDAC include suppressive immune infiltrate, scant cytotoxic T cell infiltrate, and a dense desmoplastic stroma. The inhibitory immune cells include M2 macrophages, MDSCs, and certain B cell populations which contribute immunosuppressive cytokines limiting the efficacy of antitumor immune responses by the few infiltrating CD8^+^ T cells. The desmoplastic stroma promotes angiogenesis, distorts normal architecture into a dense fibrotic matrix limiting immune infiltrate and leading to pro-tumor polarization of immune cells, as well as tumor cell invasion (reviewed in[35]). Key contributors to the deposition and development of the desmoplastic stroma are pancreatic stellate cells, or CAFs[36], and B cells[37].

There have been several attempts to improve response to conventional cytotoxic therapy by targeting the tumor microenvironment, which have been disappointing in pancreatic adenocarcinoma. The initial clinical trials combining cytotoxic therapy with checkpoint blockade or vaccine therapy have demonstrated minimal efficacy (reviewed in[35]). Attempts to target critical suppressive pathway of TGFβ in combination with radiation demonstrated a modest survival benefit, but did not result in substantial cures[1]. However, targeting of two suppressive pathways in combination with cytotoxic radiation led to impressive rates of survival. In combination with TGFβ inhibition and radiotherapy our group blocked macrophage recognition of phosphatidyl serine on apoptotic cancer cells through genetic ablation of C-MER proto-oncogene tyrosine kinase (MerTK) which redirected immunosuppressive to inflammatory cytokine production. These observations were in due in part to enhanced TNFα and reduced IL-10 production by macrophages[32].

We found that animals treated with FAP inhibitor demonstrated decreased macrophage infiltrate and enhanced disorganized collagen deposition, but after drug cessation, a reversal of phenotype with enhanced macrophage infiltrate and resolution of collagen deposition was observed. This suggests a recovery of FAP function leading to collagen cleavage and organization associated with a transient decrease in tumor size, though not due to decrease tumor cellularity (data not shown), but rather compaction of the tissue around the ECM. To determine whether duration of FAP inhibition was responsible for lack of survival benefit with radiation, we utilized mice harboring germline FAP knockout. Therapies which limit the desmoplastic stromal reaction by targeting BTK (B cells and myeloid cells) have been shown to improve cytotoxic T cell function, but did not render a significant survival advantage[37],[38], analogous to our current results from blocking FAP function.

FAP expression by CAFs has provided a potential therapeutic target in other studies. Prior publications have demonstrated that tumors grown in FAP KO mice demonstrate delayed tumor growth, but enhanced disorganized collagen deposition and p21 signaling via extracellular signal–regulated kinase (ERK) and focal adhesion kinase (FAK)[25]. Previous attempts to target CAFs by less specific αSMA knockout resulted in enhanced tumor growth and immunosuppression which could be reversed by combination with immune checkpoint blockade, anti-CTLA4[23]. Depletion of CAFs using FAP-targeting has provided mixed results with FAP directed chimeric antigen receptor (CAR) T cells demonstrating severe toxicity with cachexia and bone marrow failure due to FAP expression in adipocytes and bone mesenchymal stem cells in one model[39], while others have demonstrated tumor growth delay due to enhanced CD8^+^ T cell function and persistence associated with decreased desmoplastic stroma[19–21]. Of note, CAR T cells targeting FAP resulted in variable tumor growth delay in different tumor models, but survival data was not reported[19]. Though we demonstrate enhanced antigen-specific T cell function with FAP inhibition and knockout, this was insufficient to improve survival in combination with radiation and/or anti-PD1 antibody. We have observed no survival benefit or toxicity when CAFs were targeted with adoptive transfer of anti-βgal T cells in our *FAP*^*lacZ*^ mice. This is consistent with recent data implicating FAP+ CAFs in T cell exclusion, via TGFb signaling, and suppression of T cell proliferation [24,40]. Others have demonstrated that targeting a key mediator of stromal suppression, FAK plus gemcitabine or dual checkpoint blockade (anti-CTLA4/ anti-PD1) resulted in a modest survival improvement. However, cures were only achieved when triple therapy was utilized with FAK inhibition, anti-PD1, and gemcitabine[41]. This is in contrast to our data demonstrating targeting FAP was ineffective in combination with anti-PD1 and radiation, which may be due, in part, to FAK inhibition repolarizing tumor cytokines not seen with FAP inhibition[41]. Together, these data suggest that limiting the fibrotic stroma, enhancing T cell infiltrate and function, and cytotoxic therapy are insufficient without limiting inhibitory immune cell infiltrate and redirecting cytokine polarization.

Interpretation of our results must also be taken within the context of our experimental models. We utilized a poorly immunogenic, subcutaneous tumor cell line to model PDAC, Panc02, in order to target a single tumor with radiation. Genetically engineered mouse models (GEMMs) may better recapitulate the mutational landscape of human PDAC, but represent a more challenging model for targeted radiation given that they can develop anywhere within the pancreas, adjacent to nearby critical organs, and require fiducial placement via open laparotomy for radiation targeting. A recent publication using CAR T cells targeting FAP utilized both subcutaneously implanted tumors and tumors arising spontaneously in the Kras^G12D^:Trp53^R172H^:Pdx-1-Cre (KPC) GEMM [20]. They observed similar alterations in tumor growth, proliferation, apoptosis, stromagenesis, angiogenesis, and desmoplasia [20] suggesting that the location of the tumor did not dictate behavior or response to therapy. We found lack of efficacy in both subcutaneous Panc02/Panc02-SIY tumors, as well as orthotopic transplanted mammary carcinomas derived from the MMTV-PyMT GEMM. In addition, results of CAF depletion using GEMM demonstrate mixed results with one study demonstrating improved survival when CAF-depletion is combined with immune checkpoint blockade [42], while another demonstrates knockout of αSMA expressing cells leads to worse outcomes[23]. These conflicting data demonstrate that specificity of target influences tumor behavior and survival, and underscores the need to target specific functions of CAFs and perhaps avoid depletion of cell populations which may lead to unexpected outcomes. While inhibiting FAP function demonstrated promise in prior publications [25], we found no enhanced efficacy over radiation alone.

While we are the first to examine a selective FAP inhibitor in pre-clinical models of PDAC, our results provide room for improvement. Inhibition of FAP proteolytic activity decreased macrophage infiltrate and genetic knockout of FAP enhanced T cell infiltrate and cytotoxicity. FAP inhibition performed similarly to germline loss of FAP. Further targeting of CAFs, beyond FAP function, combined with radiation did not improve survival over radiation alone suggesting further suppressive pathways are limiting responses. The future success of FAP inhibitors may depend on the endogenous susceptibility of cancer cells to T cell mediated killing.

## Supporting information

S1 Fig No difference in hypoxia, vascularity, or CAFs. Panc02-SIY tumor bearing mice in WT or FAP KO animals, tumors harvested on day 14. a) Immunofluorescent staining for hypoxyprobe (green) and DAPI nuclear stain (blue). b) Quantification of hypoxyprobe positive area relative to DAPI positive area. Panc02-SIY tumor bearing mice in WT or FAP KO animals, tumors harvested on day 14. Panc02 tumor bearing animals in control or UAMC-1110 (UAMC) treated animals alone or following radiation (RT) at day 22 and day 43. c) Immunofluorescent staining for αSMA (green), CD31 (red), and DAPI nuclear stain (blue). d) Quantification of vessel area by CD31 positive area relative to DAPI positive area. e) Quantification of CAFs by αSMA positive area relative to DAPI positive area. f) Immunofluorescent staining for vimentin (red), Ki67 (green), and DAPI nuclear satin (blue). g) Quantification of Ki67 positive area relative to DAPI positive area. h) Quantification of vimentin positive area relative to DAPI positive area. i) Immunofluorescent staining for PDGFRα (red), cleaved caspase 3 (green), and DAPI nuclear stain (blue). j) Quantification of cleaved caspase 3 positive area relative to DAPI positive area. k) Quantification of PDGFRα positive area relative to DAPI positive area. n = 4–8 mixed gender mice/group, images representative of group and experiment. NS = not significant, ****p<0.0001.
(TIF)

S2 Fig Tumor cytokines minimally altered in FAP KO animals. Panc02-SIY tumor bearing mice in WT (WT) or FAP knockout (FAP KO) animals, randomized to receive 10 Gy x 3 tumor directed radiation (RT) days 14–16. Tumors harvested on day 23, homogenized, and evaluated for cytokine levels. n = 4–6 mixed gender mice/group. *p<0.05.
(TIF)

S3 Fig Orthotopic PyMT-MMTV tumor bearing mice in WT or FAP KO animals, randomized to receive 10 Gy x 1 tumor directed RT on day 14. Mean tumor growth curve. n = 4–8 female mice/group.
(TIF)

S4 Fig PDL1 expression in Panc02 and Panc02-SIY. a) Panc02 or b) Panc02-SIY cells treated with IFNγ or 20Gy radiation and assessed for PDL1 expression by flow cytometry 24h later.
(TIF)

## References

[1] Young KH, Newell P, Cottam B, Friedman D, Savage T, Baird JR, et al TGFβ Inhibition Prior to Hypofractionated Radiation Enhances Efficacy in Preclinical Models. Cancer Immunol Res. 2014; 10.1158/2326-6066.CIR-13-0207 25047233

[2] Zhang B, Bowerman N a, Salama JK, Schmidt H, Spiotto MT, Schietinger A, et al Induced sensitization of tumor stroma leads to eradication of established cancer by T cells. J Exp Med. 2007;204: 49–55. 10.1084/jem.20062056 17210731 PMC2118433

[3] Reits E a, Hodge JW, Herberts C a, Groothuis T a, Chakraborty M, Wansley EK, et al Radiation modulates the peptide repertoire, enhances MHC class I expression, and induces successful antitumor immunotherapy. J Exp Med. 2006;203: 1259–71. 10.1084/jem.20052494 16636135 PMC3212727

[4] Ganss R, Ryschich E, Klar E, Arnold B, Hämmerling GJ. Combination of T-cell therapy and trigger of inflammation induces remodeling of the vasculature and tumor eradication. Cancer Res. 2002;62: 1462–70. Available: http://www.ncbi.nlm.nih.gov/pubmed/11888921 11888921

[5] Lee Y, Auh SL, Wang YY, Burnette B, Meng Y, Beckett M, et al Therapeutic effects of ablative radiation on local tumor require CD8+ T cells: changing strategies for cancer treatment. Blood. 2009;114: 589–95. 10.1182/blood-2009-02-206870 19349616 PMC2713472

[6] Gough MJ, Crittenden MR, Sarff M, Pang P, Seung SK, Vetto JT, et al Adjuvant therapy with agonistic antibodies to CD134 (OX40) increases local control after surgical or radiation therapy of cancer in mice. J Immunother. 2010;33: 798–809. 10.1097/CJI.0b013e3181ee7095 20842057 PMC3563298

[7] Crittenden MR, Savage T, Cottam B, Baird J, Rodriguez PC, Newell P, et al Expression of arginase I in myeloid cells limits control of residual disease after radiation therapy of tumors in mice. Radiat Res. 2014;182: 182–90. 10.1667/RR13493.1 24992164

[8] Ene-Obong A, Clear AJ, Watt J, Wang J, Fatah R, Riches JC, et al Activated pancreatic stellate cells sequester CD8+ T cells to reduce their infiltration of the juxtatumoral compartment of pancreatic ductal adenocarcinoma. Gastroenterology. 2013;145: 1121–32. 10.1053/j.gastro.2013.07.025 23891972 PMC3896919

[9] Loeffler M, Krüger JA, Niethammer AG, Reisfeld RA. Targeting tumor-associated fibroblasts improves cancer chemotherapy by increasing intratumoral drug uptake. J Clin Invest. 2006;116: 1955–62. 10.1172/JCI26532 16794736 PMC1481657

[10] Nazareth MR, Broderick L, Simpson-Abelson MR, Kelleher RJ, Yokota SJ, Bankert RB. Characterization of human lung tumor-associated fibroblasts and their ability to modulate the activation of tumor-associated T cells. J Immunol. 2007;178: 5552–62. Available: http://www.ncbi.nlm.nih.gov/pubmed/17442937 17442937 10.4049/jimmunol.178.9.5552

[11] Zhu Y, Knolhoff BL, Meyer MA, Nywening TM, West BL, Luo J, et al CSF1/CSF1R Blockade Reprograms Tumor-Infiltrating Macrophages and Improves Response to T-cell Checkpoint Immunotherapy in Pancreatic Cancer Models. Cancer Res. 2014; 10.1158/0008-5472.CAN-13-3723 25082815 PMC4182950

[12] Hawinkels LJAC, Paauwe M, Verspaget HW, Wiercinska E, Zon JM, Ploeg K, et al Interaction with colon cancer cells hyperactivates TGF-β signaling in cancer-associated fibroblasts. Oncogene. 2014;33: 97–107. 10.1038/onc.2012.536 23208491

[13] Liao D, Luo Y, Markowitz D, Xiang R, Reisfeld RA. Cancer associated fibroblasts promote tumor growth and metastasis by modulating the tumor immune microenvironment in a 4T1 murine breast cancer model. PLoS One. 2009;4: e7965 10.1371/journal.pone.0007965 19956757 PMC2775953

[14] Mace TA, Ameen Z, Collins A, Wojcik S, Mair M, Young GS, et al Pancreatic cancer-associated stellate cells promote differentiation of myeloid-derived suppressor cells in a STAT3-dependent manner. Cancer Res. 2013;73: 3007–18. 10.1158/0008-5472.CAN-12-4601 23514705 PMC3785672

[15] Badea L, Herlea V, Dima SO, Dumitrascu T, Popescu I. Combined gene expression analysis of whole-tissue and microdissected pancreatic ductal adenocarcinoma identifies genes specifically overexpressed in tumor epithelia. Hepatogastroenterology. 55: 2016–27. Available: http://www.ncbi.nlm.nih.gov/pubmed/19260470 19260470

[16] Mazur A, Holthoff E, Vadali S, Kelly T, Post SR. Cleavage of type i collagen by fibroblast activation protein-a enhances class a scavenger receptor mediated macrophage adhesion. PLoS One. 2016;11: 1–16. 10.1371/journal.pone.0150287 26934296 PMC4774960

[17] Juillerat-Jeanneret L, Tafelmeyer P, Golshayan D. Fibroblast activation protein-α in fibrogenic disorders and cancer: more than a prolyl-specific peptidase? Expert Opin Ther Targets. 2017;21: 977–991. 10.1080/14728222.2017.1370455 28829211

[18] Kraman M, Bambrough PJ, Arnold JN, Roberts EW, Magiera L, Jones JO, et al Suppression of antitumor immunity by stromal cells expressing fibroblast activation protein-alpha. Science. 2010;330: 827–30. 10.1126/science.1195300 21051638

[19] Wang L-CS, Lo A, Scholler J, Sun J, Majumdar RS, Kapoor V, et al Targeting Fibroblast Activation Protein in Tumor Stroma with Chimeric Antigen Receptor T Cells Can Inhibit Tumor Growth and Augment Host Immunity without Severe Toxicity. Cancer Immunol Res. 2014;2: 154–166. 10.1158/2326-6066.CIR-13-0027 24778279 PMC4007316

[20] Lo A, Wang LCS, Scholler J, Monslow J, Avery D, Newick K, et al Tumor-promoting desmoplasia is disrupted by depleting FAP-expressing stromal cells. Cancer Res. 2015;75: 2800–2810. 10.1158/0008-5472.CAN-14-3041 25979873 PMC4506263

[21] Zhang Y, Ertl HCJ. Depletion of FAP+ cells reduces immunosuppressive cells and improves metabolism and functions CD8+T cells within tumors. Oncotarget. 2016;7: 23282–99. 10.18632/oncotarget.7818 26943036 PMC5029626

[22] Gottschalk S, Yu F, Ji M, Kakarla S, Song X-T. A vaccine that co-targets tumor cells and cancer associated fibroblasts results in enhanced antitumor activity by inducing antigen spreading. PLoS One. 2013;8: e82658 10.1371/journal.pone.0082658 24349329 PMC3861387

[23] Özdemir BC, Pentcheva-Hoang T, Carstens JL, Zheng X, Wu C-C, Simpson TR, et al Depletion of carcinoma-associated fibroblasts and fibrosis induces immunosuppression and accelerates pancreas cancer with reduced survival. Cancer Cell. 2014;25: 719–34. 10.1016/j.ccr.2014.04.005 24856586 PMC4180632

[24] Cremasco V, Astarita JL, Grauel AL, Keerthivasan S, MacIsaac K, Woodruff MC, et al FAP Delineates Heterogeneous and Functionally Divergent Stromal Cells in Immune-Excluded Breast Tumors. Cancer Immunol Res. 2018;6: 1472–1485. 10.1158/2326-6066.CIR-18-0098 30266714 PMC6597261

[25] Santos A, Jung J, Aziz N, Kissil J. Targeting fibroblast activation protein inhibits tumor stromagenesis and growth in mice. J Clin Invest. 2009;119: 3613–3625. 10.1172/JCI38988 19920354 PMC2786791

[26] Jansen K, Heirbaut L, Cheng JD, Joossens J, Ryabtsova O, Cos P, et al Selective Inhibitors of Fibroblast Activation Protein (FAP) with a (4-Quinolinoyl)-glycyl-2-cyanopyrrolidine Scaffold. ACS Med Chem Lett. 2013;4: 491–6. 10.1021/ml300410d 24900696 PMC4027141

[27] Jansen K, Heirbaut L, Verkerk R, Cheng JD, Joossens J, Cos P, et al Extended structure-activity relationship and pharmacokinetic investigation of (4-quinolinoyl)glycyl-2-cyanopyrrolidine inhibitors of fibroblast activation protein (FAP). J Med Chem. 2014;57: 3053–74. 10.1021/jm500031w 24617858

[28] Priebe TS, Atkinson EN, Pan BF, Nelson JA. Intrinsic resistance to anticancer agents in the murine pancreatic adenocarcinoma PANC02. Cancer Chemother Pharmacol. 1992;29: 485–9. Available: http://www.ncbi.nlm.nih.gov/pubmed/1348974 1348974 10.1007/BF00684853

[29] Lin EY, Nguyen a V, Russell RG, Pollard JW. Colony-stimulating factor 1 promotes progression of mammary tumors to malignancy. J Exp Med. 2001;193: 727–40. Available: http://www.pubmedcentral.nih.gov/articlerender.fcgi?artid=2193412&tool=pmcentrez&rendertype=abstract 11257139 10.1084/jem.193.6.727 PMC2193412

[30] Crittenden MR, Savage T, Cottam B, Bahjat KS, Redmond WL, Bambina S, et al The peripheral myeloid expansion driven by murine cancer progression is reversed by radiation therapy of the tumor. PLoS One. 2013;8: e69527 10.1371/journal.pone.0069527 23936036 PMC3723876

[31] Lykidis D, Van Noorden S, Armstrong A, Spencer-Dene B, Li J, Zhuang Z, et al Novel zinc-based fixative for high quality DNA, RNA and protein analysis. Nucleic Acids Res. 2007;35: e85 10.1093/nar/gkm433 17576663 PMC1919503

[32] Crittenden MR, Baird J, Friedman D, Savage T, Uhde L, Alice A, et al Mertk on tumor macrophages is a therapeutic target to prevent tumor recurrence following radiation therapy. Oncotarget. 2014;7 10.18632/oncotarget.11823 27602953 PMC5346667

[33] Formenti SC, Lee P, Adams S, Goldberg JD, Li X, Xie MW, et al Focal Irradiation and Systemic TGFβ Blockade in Metastatic Breast Cancer. Clin Cancer Res. 2018; 10.1158/1078-0432.CCR-17-3322 29476019 PMC5999326

[34] Niedermeyer J, Garin-Chesa P, Kriz M, Hilberg F, Mueller E, Bamberger U, et al Expression of the fibroblast activation protein during mouse embryo development. Int J Dev Biol. 2001;45: 445–7. 11330865

[35] Kunk PR, Bauer TW, Slingluff CL, Rahma OE. From bench to bedside a comprehensive review of pancreatic cancer immunotherapy. J Immunother Cancer. Journal for ImmunoTherapy of Cancer; 2016;4: 14 10.1186/s40425-016-0119-z 26981244 PMC4791889

[36] Fearon DT. The carcinoma-associated fibroblast expressing fibroblast activation protein and escape from immune surveillance. Cancer Immunol Res. 2014;2: 187–193. 10.1158/2326-6066.CIR-14-0002 24778314

[37] Gunderson AJ, Kaneda MM, Tsujikawa T, Nguyen A V., Affara NI, Ruffell B, et al Bruton tyrosine kinase–Dependent immune cell cross-talk drives pancreas cancer. Cancer Discov. 2016;6: 270–285. 10.1158/2159-8290.CD-15-0827 26715645 PMC4783268

[38] Masso-Valles D, Jauset T, Serrano E, Sodir NM, Pedersen K, Affara NI, et al Ibrutinib exerts potent antifibrotic and antitumor activities in mouse models of pancreatic adenocarcinoma. Cancer Res. 2015;75: 1675–1681. 10.1158/0008-5472.CAN-14-2852 25878147 PMC6773609

[39] Tran E, Chinnasamy D, Yu Z, Morgan RA, Lee C-CR, Restifo NP, et al Immune targeting of fibroblast activation protein triggers recognition of multipotent bone marrow stromal cells and cachexia. J Exp Med. 2013;210: 1125–1135. 10.1084/jem.20130110 23712432 PMC3674706

[40] Chakravarthy A, Khan L, Bensler NP, Bose P, De Carvalho DD. TGF-β-associated extracellular matrix genes link cancer-associated fibroblasts to immune evasion and immunotherapy failure. Nat Commun. Springer US; 2018;9: 4692 10.1038/s41467-018-06654-8 30410077 PMC6224529

[41] Jiang H, Hegde S, Knolhoff BL, Zhu Y, Herndon JM, Meyer MA, et al Targeting focal adhesion kinase renders pancreatic cancers responsive to checkpoint immunotherapy. Nat Med. 2016;22: 851–60. 10.1038/nm.4123 27376576 PMC4935930

[42] Feig C, Jones JO, Kraman M, Wells RJB, Deonarine A, Chan DS, et al Targeting CXCL12 from FAP-expressing carcinoma- associated fibroblasts synergizes with anti–PD-L1 immunotherapy in pancreatic cancer. Proc Natl Acad Sci U S A. 2013;110: 20212–20217. 10.1073/pnas.1320318110 24277834 PMC3864274

