# Supplementary figures and images for: Blockade of fibroblast activation protein in combination with radiation treatment in murine models of pancreatic adenocarcinoma
Source: PLoS One. 2019 Feb 6;14(2):e0211117. doi: 10.1371/journal.pone.0211117 (PMC6364920; doi:10.1371/journal.pone.0211117)

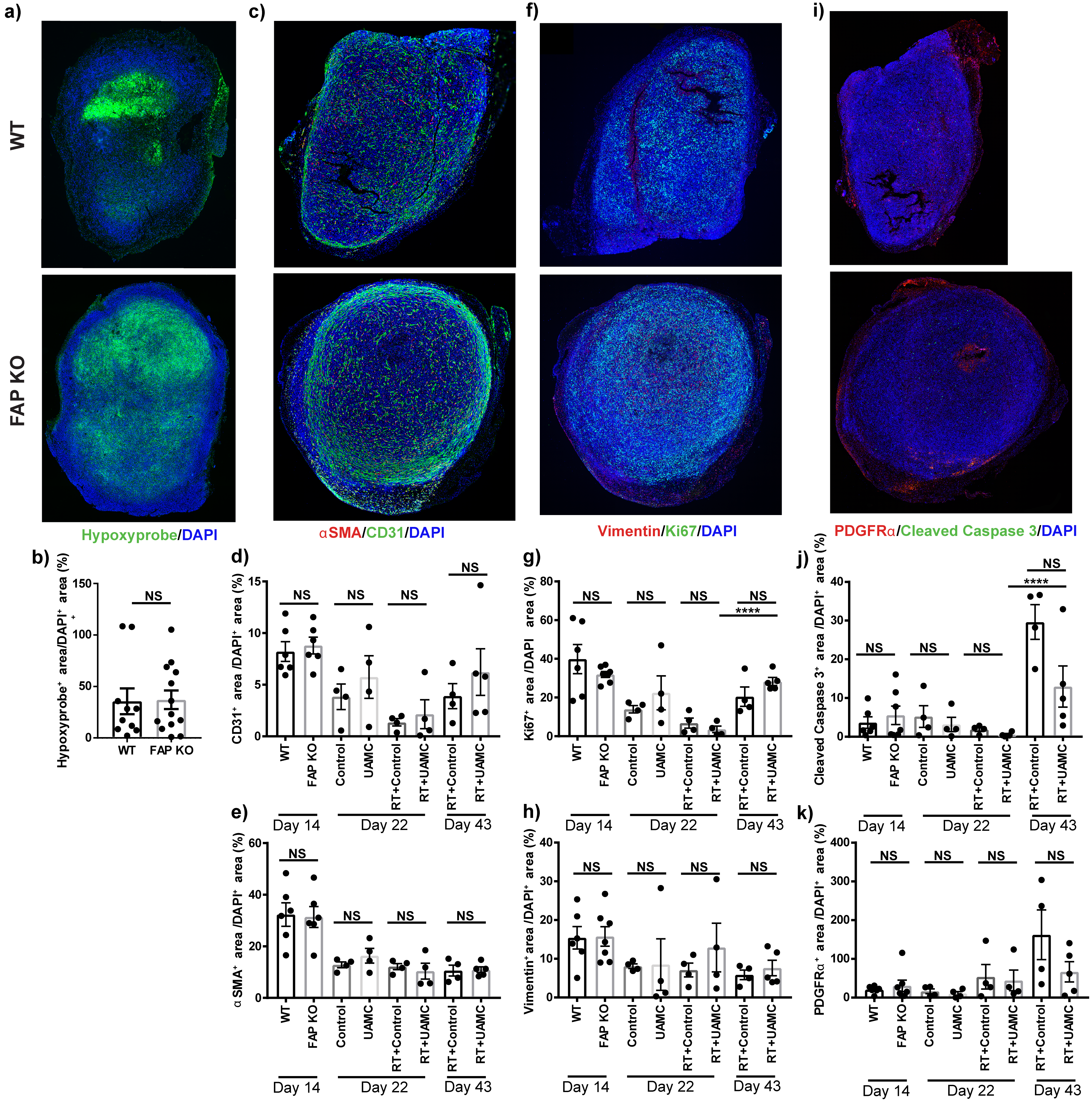

Supplement: S1 Fig — Panc02-SIY tumor bearing mice in WT or FAP KO animals, tumors harvested on day 14. a) Immunofluorescent staining for hypoxyprobe (green) and DAPI nuclear stain (blue). b) Quantification of hypoxyprobe positive area relative to DAPI positive area. Panc02-SIY tumor bearing mice in WT or FAP KO animals, tumors harvested on day 14. Panc02 tumor bearing animals in control or UAMC-1110 (UAMC) treated animals alone or following radiation (RT) at day 22 and day 43. c) Immunofluorescent staining for αSMA (green), CD31 (red), and DAPI nuclear stain (blue). d) Quantification of vessel area by CD31 positive area relative to DAPI positive area. e) Quantification of CAFs by αSMA positive area relative to DAPI positive area. f) Immunofluorescent staining for vimentin (red), Ki67 (green), and DAPI nuclear satin (blue). g) Quantification of Ki67 positive area relative to DAPI positive area. h) Quantification of vimentin positive area relative to DAPI positive area. i) Immunofluorescent staining for PDGFRα (red), cleaved caspase 3 (green), and DAPI nuclear stain (blue). j) Quantification of cleaved caspase 3 positive area relative to DAPI positive area. k) Quantification of PDGFRα positive area relative to DAPI positive area. n = 4–8 mixed gender mice/group, images representative of group and experiment. NS = not significant, ****p<0.0001. (TIF) [file pone.0211117.s001.tif]

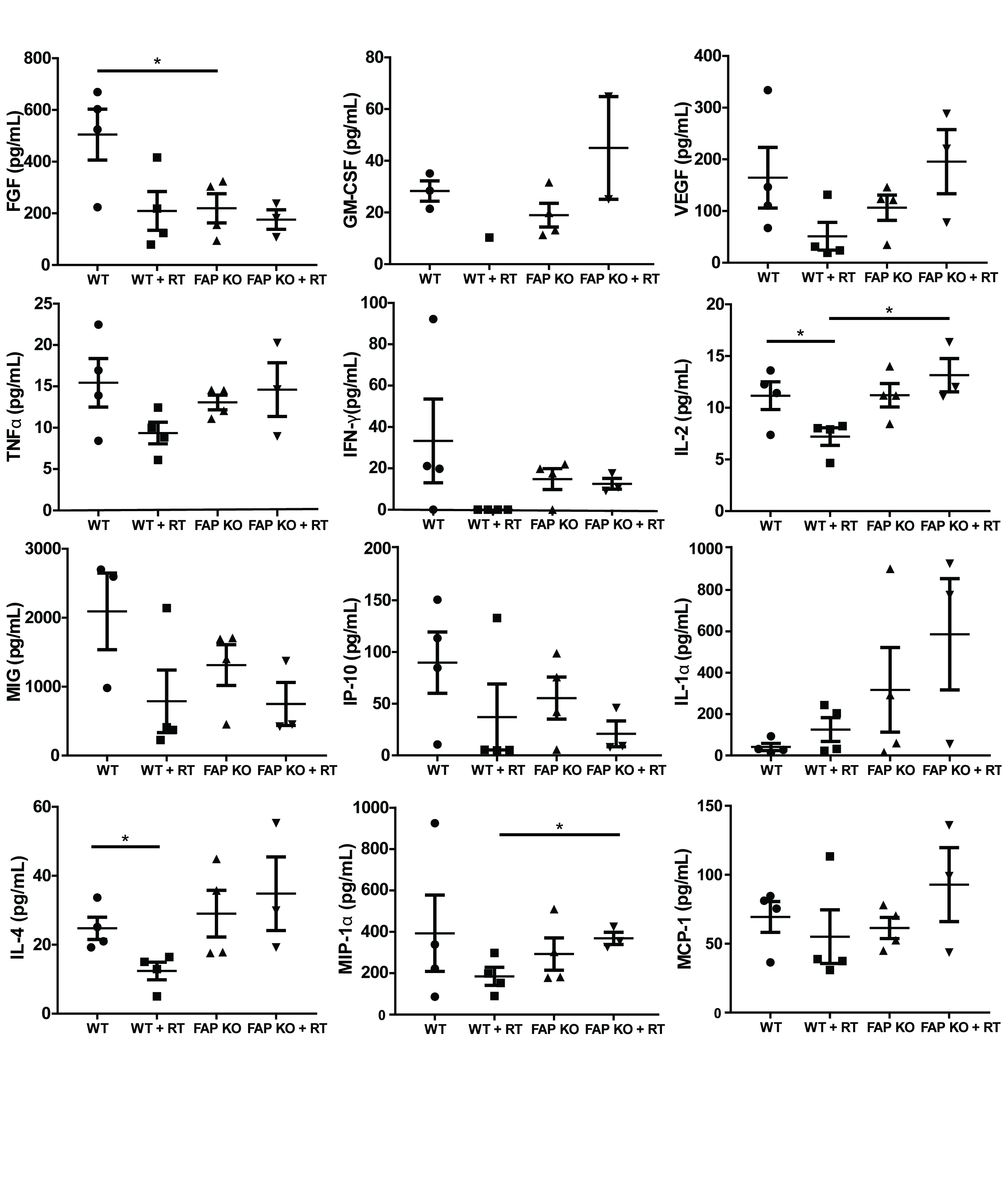

Supplement: S2 Fig — Panc02-SIY tumor bearing mice in WT (WT) or FAP knockout (FAP KO) animals, randomized to receive 10 Gy x 3 tumor directed radiation (RT) days 14–16. Tumors harvested on day 23, homogenized, and evaluated for cytokine levels. n = 4–6 mixed gender mice/group. *p<0.05. (TIF) [file pone.0211117.s002.tif]

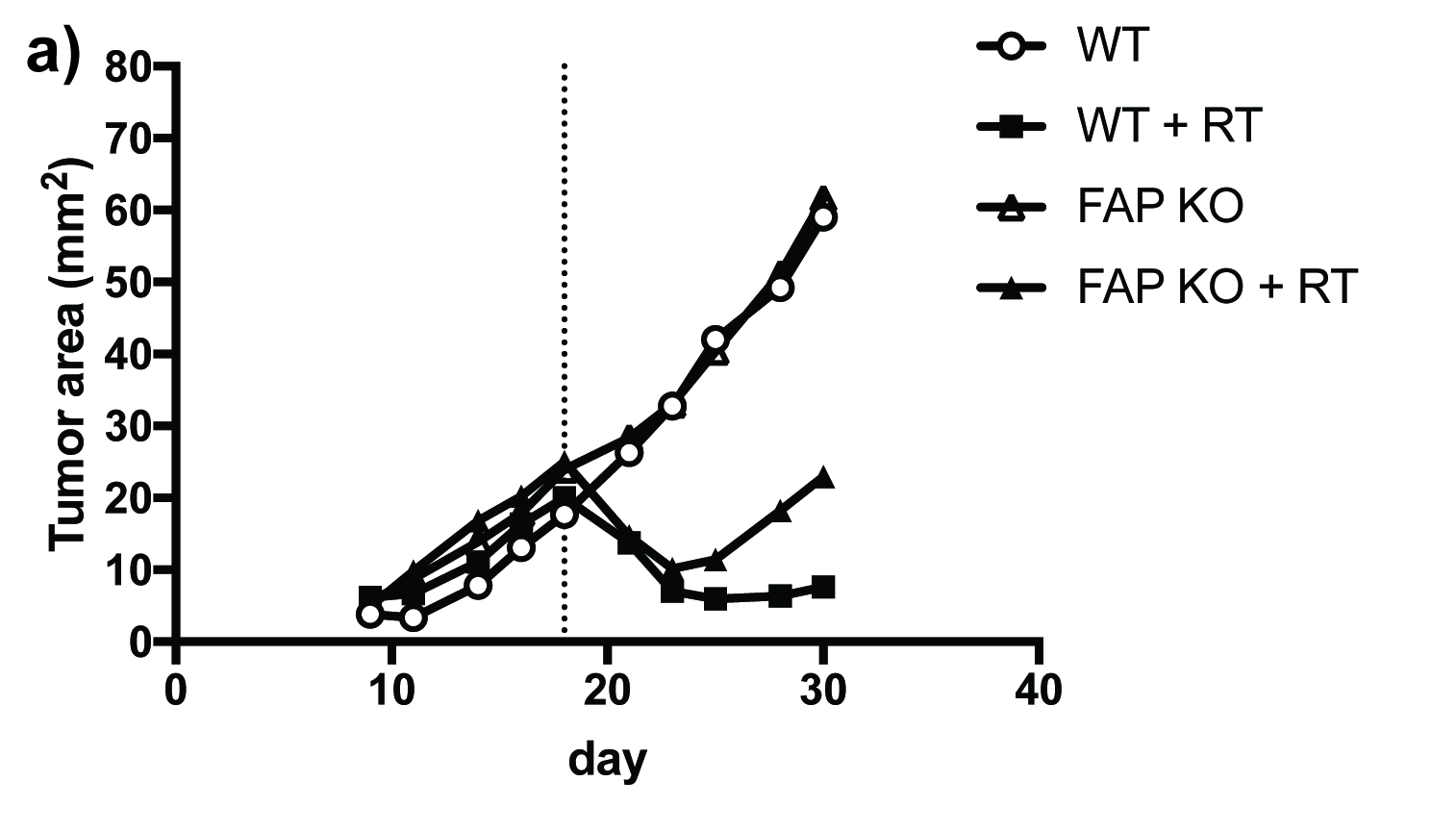

Supplement: S3 Fig — Mean tumor growth curve. n = 4–8 female mice/group. (TIF) [file pone.0211117.s003.tif]
